# CPR in medical schools: learning by teaching BLS to sudden cardiac death survivors – a promising strategy for medical students?

**DOI:** 10.1186/1472-6920-6-27

**Published:** 2006-04-28

**Authors:** Oliver Robak, Johannes Kulnig, Fritz Sterz, Thomas Uray, Moritz Haugk, Andreas Kliegel, Michael Holzer, Harald Herkner, Anton N Laggner, Hans Domanovits

**Affiliations:** 1 Department of Emergency Medicine, Medical University of Vienna, Austria Waehringer Guertel 18-20, 1090 Vienna, Austria

## Abstract

**Background:**

Cardiopulmonary resuscitation (CPR) training is gaining more importance for medical students. There were many attempts to improve the basic life support (BLS) skills in medical students, some being rather successful, some less. We developed a new problem based learning curriculum, where students had to teach CPR to cardiac arrest survivors in order to improve the knowledge about life support skills of trainers and trainees.

**Methods:**

Medical students who enrolled in our curriculum had to pass a 2 semester problem based learning session about the principles of cardiac arrest, CPR, BLS and defibrillation (CPR-D). Then the students taught cardiac arrest survivors who were randomly chosen out of a cardiac arrest database of our emergency department. Both, the student and the Sudden Cardiac Death (SCD) survivor were asked about their skills and knowledge via questionnaires immediately after the course. The questionnaires were then used to evaluate if this new teaching strategy is useful for learning CPR via a problem-based-learning course. The survey was grouped into three categories, namely "Use of AED", "CPR-D" and "Training". In addition, there was space for free answers where the participants could state their opinion in their own words, which provided some useful hints for upcoming programs.

**Results:**

This new learning-by-teaching strategy was highly accepted by all participants, the students and the SCD survivors. Most SCD survivors would use their skills in case one of their relatives goes into cardiac arrest (96%). Furthermore, 86% of the trainees were able to deal with failures and/or disturbances by themselves. On the trainer's side, 96% of the students felt to be well prepared for the course and were considered to be competent by 96% of their trainees.

**Conclusion:**

We could prove that learning by teaching CPR is possible and is highly accepted by the students. By offering a compelling appreciation of what CPR can achieve in using survivors from SCD as trainees made them go deeper into the subject of resuscitation, what also might result in a longer lasting benefit than regular lecture courses in CPR.

## Background

Sudden cardiac arrest is a major public health problem. The use of automatic external defibrillators (AED) not only by security personal, but also by concerned laymen and their relatives has become an important part of emergency medical systems [1]. Still, the outcome of an out-of-hospital cardiac arrest is bad, mostly due to lack of laymen's skills in basic life support (BLS) [2]. Especially sudden cardiac death (SCD) survivors have a high risk for a re-arrest, because not all of them receive a sufficient prophylactic strategy in case of recurrence [3]. As ventricular fibrillation [4] is still the major primary rhythm in cardiac arrest [5,6], nowadays available AEDs can be used especially by the trained and even untrained laymen [7]. This is important due to the fast declining success rates for restoration of spontaneous circulation after longer periods of ventricular fibrillation. Still the discussion about the fastest way of getting the AED to the patient – especially at their homes – remains unsolved. Therefore, providing patients with AEDs at their homes might be a promising strategy for increasing the success rates after sudden cardiac arrest [8].

On the other hand, there is the need of teaching medical students in BLS and the use of AEDs. Though being a real important issue, there is still too less attention within the curriculum at medical universities for teaching life support skills in an attractive way. Therefore we offered our students a new teaching strategy called "learning by teaching", where medical students trained SCD survivors at their homes in BLS and the use of an AED. The subject of cardiopulmonary resuscitation (CPR) and defibrillation is undeniably important in the training of medical students and medical doctors. Since there was no mandatory course in CPR in the current curriculum at our medical school, only those students that voluntarily enrolled in our elective CPR course received this type of problem based learning courses. Thereby we thought on the one hand to provide patients with this important information about life support issues and on the other hand these soon-to-be medical professionals to learn more about their patients and especially to better learn by teaching BLS and the use of an AED.

The goal of this specific investigation was to compare the evaluation given by both the trainer and the trainee in order to further develop and test this teaching strategy that is using medical students. We focussed on effectiveness, compliance and quality of the course. We assumed if both evaluations match, both tested groups might have a benefit and therefore our strategy might work.

## Methods

Medical students trained in BLS and the use of an AED have been asked to participate in special courses provided by staff members of our emergency department. Through these teaching sessions students were prepared in how to contact SCD survivors and their families and how to provide them with an AED and sufficient knowledge about how to use the AED including BLS at the patient and relatives desire. Prospectively developed evaluation forms for the students and the families were compared retrospectively with questions focusing on the effectiveness, compliance and quality of the course. In addition students and families had enough space to freely comment on the new form of "learning by teaching".

### Participants

#### The patients (trainees)

The participating patients were randomly chosen out of a cardiac arrest database of a Department of Emergency Medicine, where patients suffering from cardiac arrest are registered since 1991 following the Utstein guidelines [9]. We only included patients who survived without any neurological failure and who were fully reintegrated into their normal lives. We excluded patients under 18 years of age at the time of cardiac arrest, patients that received an implantable cardioverter-defibrillator, patients suffering from severe or incurable illness, patients with psychiatric illnesses, patients with "Do-not-resuscitate" decisions and patients who seemed to be mentally or physically not able to perform BLS correctly, e.g. disabled patients. Previous studies have shown that these patients should have a risk of re-arrest of 25–50% [10-12].

Once a patient was eligible, a medical student made contact to see if he/she was still interested in participating in this trial. At this time an appointment was made when and where the training should take place. In order to enhance the compliance and acceptance of the training, it was planned to meet the patients and their relatives at their homes. The patients and their spouses were asked to invite people who share most of their time with them, e.g. children or co-workers.

#### The students (trainers)

Since laymen not necessarily needed to be trained by professionals [13] in order to provide CPR correctly and too less teaching personal would have been available via our staff, trainers were recruited out of motivated medical students, who had been trained in basic and advanced life support according to current guidelines [14]. Special attention was paid to in how to handle the provided AED and to follow the safety guidelines.

### Teaching the students (trainers)

In order to reach the best possible outcome for both the student and the trainee, classes had to be held for the students where they should receive all relevant information about CPR and the AED. Therefore we decided to provide an elective course for interested and motivated students. This course lasted for 2 semesters. The students had to repeat the basics about anatomy and physiology as far as cardiac arrest was concerned. Later, the principles of a cardiac arrest were explained, including the impact on the organs, especially the brain. In addition all had the chance to talk with a SCD survivor. The latest studies concerning CPR and defibrillation were presented, mostly by a student, who thereby had the chance to practice rhetorical skills. Furthermore, the used AED was presented by an employee of Laerdal Corporation (Rorarco GmbH, Vienna, Austria) that provided the AEDs. Again, the students had enough time to state their questions and to discuss the apparatus and its usage.

### Teaching the patients (trainees)

Unconsciousness, respiratory and cardiac arrest were explained, and basic physiological principles, necessary for the understanding of an AED and CPR in general, were exemplified. Later the AED was introduced and the major functions were explained and demonstrated. Also the need of correct placement of the pads and the importance of external chest compressions was stressed. At all times, the trainees could ask questions. After those brief theoretical explanations, practical training was conducted. We considered that as AEDs are more or less self-explaining, learning by doing would be the best way to teach CPR and BLS. All participants were encouraged to practice as long and as often until they felt confident about BLS and defibrillation using an AED. For training purposes, manikins (Little Anne™ CPR Manikin, Laerdal Corporation, Rorarco GmbH, Vienna, Austria) and a training defibrillator (Laerdal Forerunner Trainer™ and HeartStart 1 Trainer™, Laerdal Corporation, Rorarco GmbH, Vienna Austria) were used. As the usage differs between various AEDs [15], we decided to use only one model for our study, in order to receive data that can be compared.

### Evaluation

Data collection was done immediately after the course, simultaneously by the trainer and the trainees. Both had to fill a brief survey. The questions covered the length of the course, quality, diagnosis, failures, competence of the trainer, and most of all: the benefit for the trainees, e.g. if the trainees think to be able to deliver a shock within 2 minutes.

We decided on 3 different categories to be covered by the survey, namely "Use of AED", "CPR-D" and "Training". Each category consisted of various questions that had to be answered with grades ranging from 1("strongly agree") to 5("strongly disagree"). Each question was therefore posed in a way the trainers/trainees can easily answer it. In some questions, we summed up "strongly agree" and "agree", to "Yes". Answers from the patient's questionnaire were compared to answers from the trainer's questionnaire. Both the trainer and the trainee were asked similar questions about their capability and the quality and effectiveness of the course (Tab.1). Therefore in case of concordance with regards to the answers, we think to be able to draw conclusions about the benefit from our courses for the trainers and trainees.

Furthermore, the interviewee could state his opinion in free questions that had not to be rated. In these questions, we provided an opportunity to give advice in how to improve the course, or just to say what was liked best/worst. This opportunity was used frequently and gave us some good hints that will be utilised in next year's elective courses.

### Statistics

Data are presented as median and interquartile range (IQR) of the answers given, when grades from 1 to 5 were possible answers. As a consequence of data characteristic we performed non-parametric analyses. We tested whether pair wise assessment of trainers and trainees was different using a Wilxoxon test. For questions where linkage of trainers and trainees was possible only at the trainer-group level we used the median value of the groups for comparison. Furthermore, kappa with the 95% confidence interval was used to measure the agreement between the participants. A two-sided p-value < 0.05 was considered statistically significant. Statistical analysis was performed with SPSS version 12.0.

## Results

Out of 67 questionnaires filled by the trainers, 35 were excluded due to not being able to match as out of 180 questionnaires filled by trainees, 64 had to be excluded for the same reason. So all in all, there were 32 questionnaires filled by trainers and 116 questionnaires filled by trainees left for evaluation.

### Demographics

Out of 116 trainees, 51 (44%) were male, 65 (56%) were female. Of all participants 2.6% actually had resuscitated a person before.

The mean age of the trainers was 26 (minimum 22, maximum 32), 63% being female, 31% male (6% missing data). We had 56% clinical students and 34% pre-clinical students (10% missing data). Considering per-course training, 53% already participated in a first-aid course, and 47% had experiences with a defibrillator before. Advanced cardiac life support training has been provided to 78% of the participants before enrolling in our course.

The training lasted 1 hour 54 minutes in average (minimum 45 min., maximum 3 hours). In 78% the training took place at the patient's home, in 6% at the hospital, and in 16% in another place, e.g. at work. One training group consisted of 4 trainees in average (minimum 2, maximum 9) and 1 trainer.

### Use of an AED

**Figure 1:**
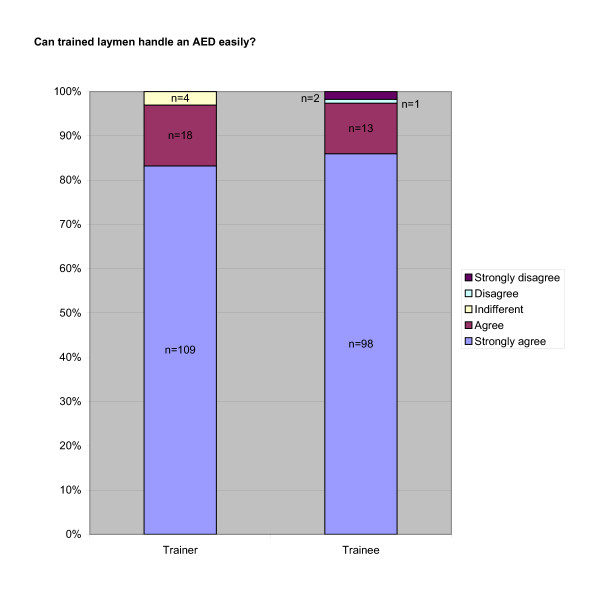
**Can trained laymen easily handle an AED?**. The trainer (student) rated how easy it was for the trainee (patient) to use an AED. The trainee (patient) rated how easy it was for him to use an AED. ("The participants believe that the usage of an AED is easy."/"I believe that the usage of an AED is easy.").

Out of 127 trainees, 87 (69%) were rated by their trainers as that they can deal with failures/disturbances of the AED by themselves (median score 2, IQR 2–3). On the other hand, 95 (85%, median score 1, IQR 1–2) out of 112 trainees thought that they can provide CPR correctly even in case of failures or disturbances. Comparing the assessment of trainees and trainers, we found a statistically significant p-value (p < 0.001). When asked about on whom they would use the AED, 107 (96%, median score 1, IQR 1-1) out of the 112 trainees will use their skills in case one of their relatives goes into cardiac arrest, 106 (95%, median score 1, IQR 1-1) will even use them on victims, who they don't know. After the course, most trainees were convinced that the use of an AED is easy and also their trainers thought that they can easily handle an AED (Fig. 1). Nevertheless, the p-value failed to reach statistical significance (p = 0.135).

### CPR course

**Figure 2:**
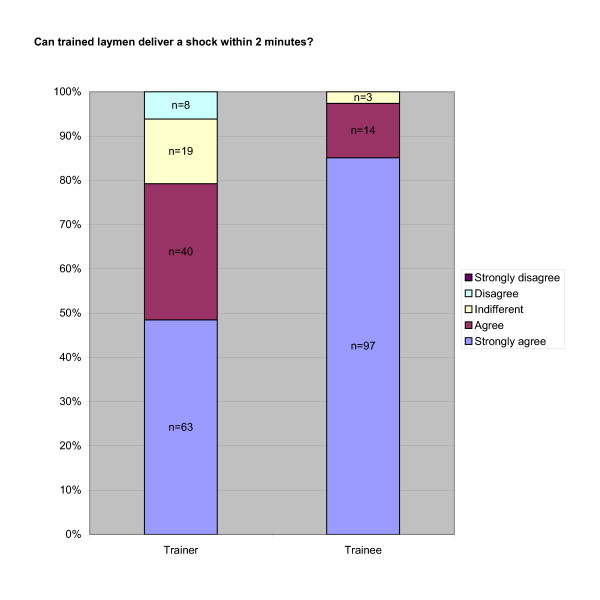
**Can trained laymen deliver a shock within 2 minutes?**. The trainer (student) rated if the trainee (patient) would be able to deliver a shock within 2 minutes in case of a cardiac arrest. The trainee (patient) rated how confident he felt about delivering a shock within 2 minutes in case of a cardiac arrest. ("The participant would deliver a shock within 2 minutes in case of an emergency."/"I would be able to deliver a shock in case of an emergency.").

**Figure 3:**
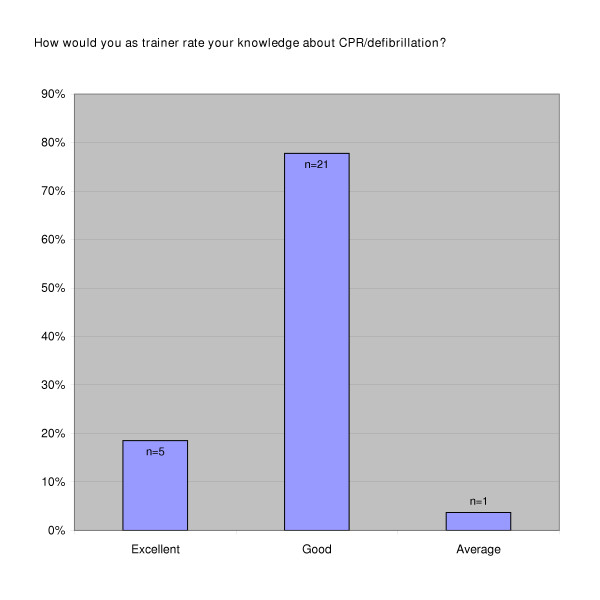
**How would you as trainer rate your knowledge about CPR/defibrillation?**. Students as trainers in a CPR course for Cardiac Arrest Survivors were asked about their knowledge about the topics of CPR and defibrillation after the course. Excellent – My knowledge about CPR and defibrillation is excellent compared to other students. Good – My knowledge about CPR and defibrillation is good compared to other students. Average – My knowledge about CPR and defibrillation is average compared to other students.

When asked about the use of an AED in an emergency situation, 103 (79%, median score 2, IQR 1–2) of the 130 trainees were believed to be able to deliver a shock within 2 minutes in case of an emergency whereas 111 (97%, median score 1, IQR 1-1) of the 114 trainees believed that they can use the AED in an emergency correctly, namely to deliver a shock within 2 minutes (p < 0.001; Fig. 2). Out of 110 trainees, 80 (73%, median score 2, IQR 1–3) felt confident to diagnose a cardiac arrest correctly whereas out of 129 trainees who were rated by their trainers, 110 (85%, median score 2, IQR 1–2) were believed to diagnose cardiac arrest correctly (p = 0.380). After the course, out of 27 trainers who have filled this question, 26 (96%, median score 1, IQR 1-1) rated their knowledge about CPR and defibrillation either "Excellent" or "Good" (Fig. 3).

### Training

In this section we focused on the effectiveness of training the students by our curriculum. All in all, 26 (96%, median score 2, IQR 2-2) of 27 of the trainers felt to be well prepared, on the other hand 109 (96%, median score 1, IQR 1-1) of 113 trainees mentioned their trainer to be competent (p = 0.097, Kappa = -0.037; 31% failed matching and have been excluded), and 106 (94%, median score 1, IQR 1-1) of 113 trainees thought that their trainer answered all their questions sufficiently. 29 (91%, median score 1, IQR 1–2) of 32 trainers considered their trainees to be interested in the topic and therefore asked a lot of questions.

Beside these 3 categories, there were also free questions that could, but not necessarily had to be answered. We picked 2 questions and some typical quotations from students who participated in the training program to illustrate the scope of responses (Tab. 3).

## Discussion

In this study, we found that "learning by teaching" with the most innovative feature by using survivors form SCD as trainees – a compelling appreciation of what CPR can achieve – improves students' confidence in their knowledge about CPR and the use of an AED. Also laymen welcome the training in BLS including the use of an AED by medical students. This strategy of "learning by teaching" is feasible and might provide a more thorough knowledge about life support to the students as trainers, because they had to deal with CPR in a different way than usual and got a deeper insight in the topic. This new strategy to use motivated medical students to teach CPR and using an AED to laymen has never been described before. Also, the benefits for both sides, the patient who is trained and the student who learns by teaching are obvious, but have not been proven so far.

Developing training methods and strategies to reach the optimum level of education and to reduce peoples concerns about using an AED and CPR must be a high goal. Only through this way it is possible to increase the survival rates of patients in ventricular fibrillation, which is still the majority first ECG-rhythm seen in cases of out-of-hospital cardiac arrest [16,17].

The benefit for the patient as trainee is high: out of 116 trainees who filled the questionnaire, 108 (93%, Median 1, IQR 1–2) rated the benefit of the course for themselves either 1 or 2. As the majority of trainees never got in touch with the topic of resuscitation, this training taught them the chain-of-survival concept including the use of an AED.

On the student's side, taking part in this new problem-based-learning program made them deal with scientific research papers, practicing their rhetorical and teaching skills and most important: it made them go deeper into the subject of resuscitation and defibrillation, which has been our main goal.

We presume that the better the patients were trained in CPR and the better they rated their trainers, the more benefit the students might have had from our "learning-by-teaching" program, as they had to be well prepared to get a good evaluation by their trainees. This was the case. Trainers who felt to be well prepared also received good ratings by their trainees. Therefore we can also the conclusion that the benefit from teaching CPR is high (Fig. 3). Not a single student rated his knowledge about CPR/defibrillation either "Bad" or "Very bad" after the course. In addition the students' feedback has shown us that our curriculum is highly useful.

Of course, this new teaching strategy still has its weaknesses: As this course was not mandatory, the participating students already had an interest in this topic and were highly motivated to participate. Another point may be, that the place chosen for the training – in most cases the patient's home – appears not to be optimal, because there were sometimes minor disturbances. Still, we think that that the training needs to take place in a realistic surrounding where cardiac arrest does happen, and that is the patient's home.

Talking about the evaluation of the questionnaires, we had to exclude a big part of the questionnaires due to the reason that they couldn't be matched. That was because during the project we further developed the type of questionnaire as we found the old one to be impracticable. Therefore some important questions slightly changed over the time and were excluded from evaluation. Also not every trainee who participated in the course filled a questionnaire, but nevertheless was rated by a trainer. So in some questions, there were more trainees rated by their trainers than trainees who filled the questionnaire what also affected the evaluation. The assessment of acquisition of CPR skills was subjective. This has been shown to be unreliable. But still so with think, that a subjective assessment does have it's value and therefore our project of "learning by teaching" to survivors of SCD its justification to serve as an innovative example for a curriculum at medical Universities.

Another point is that in a large number of questionnaires some questions remained unanswered, that means that the person that filled it – mostly a trainee – skipped this question. We think that is mostly because of the quantity of the questions that were stated. By reducing the size of the questionnaire we should be able to reduce the number of unanswered questions.

The great majority of studies that have been conducted about this topic was dealing with the improval of the BLS skills of medical students or laymen by lecture courses [18-20]. In our study, we wanted to show that learning by teaching BLS also is a promising strategy. Although there was one study that showed that there was no statistically significant difference in an objective test about diabetes when nursing students had been taught the topic either via lecture course or via problem based learning curriculum [21], we think that when it comes to practical skill acquisition, a problem based learning strategy is more promising and has a lasting effect. Another recent study similar to ours shows that the acceptance of problem based learning sessions in emergency medicine is high and therefore proves our theory [22].

When looking at our results, we think that establishing a problem-based learning program in existing curricula of medical schools will improve the knowledge about CPR and even more important the confidence to deal with a cardiac arrest in the graduates. Still, this has yet to be researched, as there are no long-term observation studies available and there hasn't been a study that directly compares our program with a common CPR course.

## Conclusion

All in all we could prove that learning by teaching to survivors of SCD – a compelling appreciation of what CPR can achieve – is a strategy that works. Also, we think that the benefit for the students is bigger and will last longer than any benefit from a regular CPR course.

## Competing interests

Financial competing interests

Funding of this trial was done by Austrian Nationalbank Anniversary Foundation, Bjoern Steiger Foundation Germany, Lions Club Vienna, Medical Scientific Foundation by the Mayor of Vienna, Ministry of Science and Traffic, Austria for EC program, Philips Medical Systems and The Laerdal Foundation for Acute Medicine.

Non-financial competing interests

There weren't any non-financial competing interests in this particular study.

## Authors' contributions

OR participated in the design and coordination of the study, carried out teaching of students in CPR, participated in the creation of questionnaires, obtained and analysed the data, interpreted the findings, drafted the manuscript and had full access to all the data in the study. JK participated in the design of the study, carried out teaching of students in CPR, obtained and analysed the data, interpreted the findings, and helped to draft the manuscript. FS conceived, designed and coordinated the study, developed the problem based learning curriculum, carried out teaching of students in CPR, managed the cardiac arrest survivors database of our emergency department, participated in the creation of questionnaires, interpreted the findings, helped to draft the manuscript, had full access to all the data in the study and has the final responsibility for the decision to submit for publication. TU participated in the design and coordination of the study, developed the problem based learning curriculum, carried out teaching of students in CPR, managed the cardiac arrest survivors database of our emergency department, participated in the creation of questionnaires, interpreted the findings and contributed to the writing of the article. MH participated in the design and coordination of the study, developed the problem based learning curriculum, carried out teaching of students in CPR, participated in the creation of questionnaires, interpreted the findings and contributed to the writing of the article. AK participated in the design and coordination of the study, carried out teaching of students in CPR, interpreted the findings and helped to draft the manuscript. MH carried out teaching of students in CPR, obtained and analysed the data, performed the statistical analysis, interpreted the findings and contributed to the writing of the article. HH participated in the design of the study, carried out teaching of students in CPR, analysed the data, performed the statistical analysis, interpreted the findings and contributed to the writing of the article. ANL participated in the design and coordination of the study, developed the problem based learning curriculum, carried out teaching of students in CPR and contributed to the writing of the article. HD participated in the design and coordination of the study, developed the problem based learning curriculum, carried out teaching of students in CPR, interpreted the findings and contributed to the writing of the article.

All authors read and approved the final manuscript.

## Pre-publication history

The pre-publication history for this paper can be accessed here:



## Figures and Tables

**Table 1:** Examples for corresponding questions in trainer's and trainee's questionnaires about BLS training, CPR and AEDs.

**Questions about the training**	
*Trainer (student)*	*Trainee (Sudden Cardiac Death Survivor)*
The preparation in the elective course was good.	The trainer appears to be competent.
The participants were interested and stated a lot of questions.	All important questions were answered sufficiently.
The trainee group was congenial.	The trainer was congenial.

**Questions about the AED**	

*Trainer (student)*	*Trainee (Sudden Cardiac Death Survivor)*
The participants can deal with failures/disturbance by themselves.	I can provide CPR-D correctly even in case of failures/disturbance.
The participants believe that that the usage of an AED is easy.	I believe that the usage of an AED is easy.

**Questions about the CPR course**	

*Trainer (student)*	*Trainee (Sudden Cardiac Death Survivor)*
The participants will be able to deliver a shock within 2 minutes.	I can use the AED correctly in case of an emergency.
The participants diagnose cardiac arrest correctly.	I recognize the symptoms of cardiac arrest in others.

**Table 2:** Courses and training the students had before enrolling in our curriculum.

**Course or training**	**Yes**	**No**
First Aid Course	17 (53%)	25 (47%)
Megacode Training	25 (78%)	7 (22%)
CPR Training	22 (69%)	10 (31%)
ECG Course	2 (6%)	30 (94%)
AED Course	15 (47%)	17 (53%)
Practical Training in Internal Medicine	1 (3%)	31 (97%)

First Aid course: 16-hour course in First Aid, including BLS, but also basic wound treatment.

CPR training: several training sessions in BLS with a manikin and an AED.

AED course: introductory course to the use of an AED in an emergency situation.

Megacode training: Advanced Cardiac Life Support training, that follows BLS training.

ECG course: training sessions in interpreting an ECG and diagnosing.

Practical training in Internal Medicine: 4 week clinical course in Internal Medicine.

**Table 3:** Two example questions from the student's questionnaire and some typical quotations from students who participated in the training program.

**Questions**	**Students' answers**
"What did you find uncommon?"	"...to stand in front of a group, explaining and speaking to them and they listened to me. It really was quite an experience for me!"
	"...to take center stage as a teacher and to talk about CPR to a group that really took an interest in the topic."
"What did you like best?"	"I was really glad that I could pass on some of my knowledge to the patients and through this, helping to get the subject over to the public."
	"The trust the group put in me. That really made me feel confident that I taught them CPR well."
